# Effects of artificial shade on the performance and water intake of feedlot beef cattle in tropical environments

**DOI:** 10.1007/s11250-026-05306-0

**Published:** 2026-09-17

**Authors:** Marcos Chiquitelli Neto, Rafael de Arruda Saron, Karime Chukr Bazzo, Diogo Tiago da Silva, Sheila Tavares Nascimento

**Affiliations:** 1 Biology and Animal Science Department, Sao Paulo State University, Ilha Solteira, SP Brazil; 2 Department of Animal Science, State University of Maringá, Maringá, PR Brazil

**Keywords:** Animal biometeorology, Nellore, Thermal comfort, Welfare

## Abstract

Heat stress is one of the main challenges for animal production systems in tropical regions. One of the ways to mitigate the negative effects of thermal stress is to provide artificial shading structures. This work experimentally evaluates the effects of artificial shading on the productivity and water consumption of finishing beef cattle. The experiments were conducted in two locations: the Amazon Forest region (Mato Grosso State, Brazil) and the Atlantic Forest region (São Paulo State, Brazil). In both experiments, the animals were equally divided into groups with and without access to shade, totaling 997 animals. Beef cattle without access to shade drank 16.2% more water. Accumulated weight gain and finishing fat were higher in animals with access to shade (90.77 vs. 85.99 kg in the first experiment, *p* < 0.05; 88.85 vs. 84.75 kg in the second experiment, *p* < 0.05). Carcass yield was similar for both groups. In conclusion, artificial shading for feedlot beef cattle led to a reduction in water consumption (consistent with improved thermal comfort) and an increase in animal performance (greater cumulative weight gain), improving overall animal welfare within tropical feedlot systems.

## Introduction

Heat stress is one of the main challenges for livestock systems in tropical and subtropical regions, as it negatively affects animal comfort, welfare, and performance (Cooke et al. 2020). Cattle raised in Brazilian tropical regions are subjected to intense solar radiation—up to 800 W/m²—making them particularly susceptible to thermal stress (Silveira et al. 2020).

Under heat stress, cattle activate a range of adaptive thermoregulatory mechanisms encompassing behavioral, physiological, neuroendocrine, and molecular responses (Gaughan et al. 2021). Typically, such stress leads to reduced feed intake and elevated body temperature, respiratory rate, increased sweating, protein metabolism, and water intake. A practical strategy to mitigate the adverse effects of heat stress in cattle raised under field conditions is the use of artificial shade structures, which reduce exposure to direct solar radiation and enhance animal comfort, welfare, and performance (Maia et al. 2020).

According to Park et al. (2020), shaded feedlot systems improve animal welfare by decreasing the thermal radiation load on cattle and, consequently, alleviating heat stress. For instance, providing shade can increase average daily weight gain by approximately 90 g per animal (Palhares et al. 2023). Beef cattle feedlots are typically located in open areas, where soil temperatures can exceed 50 °C during the hottest months, and diets rich in grains or concentrates further elevate metabolic heat production after feeding and feeding behavior may shift toward cooler periods of the day under hot conditions (Wankar et al. 2024).

Rising consumer awareness of animal welfare has fostered a distinct market segment, driven by the perception that products from well-treated animals are safer, healthier, and more ethically produced, prompting a willingness to pay higher prices (Lotti & Ferrarezi Junior, 2023). Therefore, this study aimed to evaluate the effects of artificial shading on water consumption, weight gain, and carcass characteristics of feedlot beef cattle raised in tropical regions.

## Materials and methods

### Experiments

Two experiments were conducted to evaluate the effects of artificial shading on feedlot beef cattle. The first experiment was carried out during spring (August to October 2002; 103 days) with 300 Nellore heifers (Table 1) housed in a commercial feedlot located in the Amazon Rainforest region, Tangará da Serra, Mato Grosso State, Brazil (14°38′03″ S, 57°29′04″ W). The local climate is classified as Aw (tropical climate with a dry winter) according to the Köppen system (Alvares et al. 2013). Animals were randomly allocated to six paddocks (25 × 70 m each), three without shade and three equipped with a 70% blockage shade cloth, providing 1.5 m² of shaded area per animal as recommended by Hagenmaier et al. (2016) and 0.7 m of linear feeder space. The diet (Table 2) consisted of 35% roughage and 65% concentrate on a dry matter basis and was offered three times daily.

The second experiment was conducted during summer (October to December 2003; 90 days) with 697 steers (Table 1) in a commercial feedlot located in the Atlantic Forest region, Altair, São Paulo State, Brazil (20°30′05″ S, 49°03′15″ W). The local climate is also classified as Aw (tropical climate with a dry winter) according to the Köppen system (Alvares et al., 2013). Animals were divided into two groups according to initial body weight (lighter and heavier) and were randomly assigned to four paddocks (25 × 60 m each), two with shade and two without. Shaded paddocks were covered with a 70% blockage shade cloth, providing 1.5 m² of shaded area per animal as recommended by Hagenmaier et al. (2016) and 0.7 m of linear feeder space. The diet (Table 2) consisted of 50% roughage and 50% concentrate on a dry matter basis and was also offered three times daily.

**Table 1:** Characteristics of the animals in Experiments I and II

Experiment	Treatment	Animal category	Breed composition	Initial weight (kg)	Number of animals
I (Amazon region)	Shade	Heifers	Nellore	267.7 ± 13.5	150
	No shade	Heifers	Nellore	266.3 ± 8.4	150
II (Atlantic Forest region)	Shade	Lighter steers	Nellore / Crossbred	348.5 ± 16.2	178
	No shade	Lighter steers	Nellore / Crossbred	349.9 ± 17.0	178
	Shade	Heavier steers	Nellore / Crossbred	387.7 ± 22.3	170
	No shade	Heavier steers	Nellore / Crossbred	386.7 ± 20.7	171

**Table 2:** Dietary ingredients used in each Experiment

Experiment I		Experiment II	
Component	Quantity (%)	Component	Quantity (%)
Sugar cane	35.00	Napier’s Silage	39.00
Sorghum	33.20	Citrus Pulp	25.50
cottonseed	14.00	Cotton Seed	11.40
Soybean Husk	13.71	Sugar cane	11.00
Urea	1.27	Corn	9.10
Soy bran	1.00	Cotton Bran	1.48
Calcite Limestone	1.00	Mineral Salt	0.89
White Salt	0.34	Urea	0.71
Bicalcium Phosphate	0.21	White Salt	0.66
Ammonium Sulfate	0.19	Premix	0.26
Premix	0.08	-	-

### Meteorological measurements

Meteorological variables were recorded on an hourly basis in both shaded and sun-exposed areas. Air temperature and relative humidity were measured using a psychrometer, while black-globe temperature was determined with a 15 cm hollow copper sphere positioned at a height of 1.40 m above the ground. In Experiment 1, artificial shading was provided using a Sombrax^®^ shade screen (Nortene), whereas in Experiment 2, Sombrite^®^ shade structures (Sombrite brand) were employed.

The THI was calculated using the formula:

$$\:THI=\:0.8\:\times\:\:TA\:+\:(RH/100)\:\times\:\:(T\:A\:-14.4)\:+\:46.4$$

Where:

TA is the air temperature, ºC.

RH is the air relative humidity, %.

### Performance measurements

Accumulated weight gain (final weight − initial weight) was determined at the end of each experiment after a 12-hour fasting period. Finishing fat and carcass yield were assessed at the slaughterhouse. In Experiment 1, finishing fat was visually evaluated on carcasses refrigerated for 24 h, using the scoring system described by De Felicio (1981): 1 (no fat), 2 (1–3 mm), 3 (3–6 mm), 4 (6–10 mm), and 5 (greater than 10 mm of fat). Carcass yield in Experiment 1 was calculated as the hot carcass weight (after removal of blood, hide, head, viscera, limbs, and other non-carcass components) divided by the live animal weight.

### Water consumption

In Experiment 2, water consumption was recorded hourly in all paddocks between 08:30 and 17:00 by measuring the water volume in the trough before and after each drinking event.

### Data analysis

Normality and homoscedasticity tests were performed to apply the analysis of variance.

Data were analyzed using analysis of variance (ANOVA) with the Statistical Analysis System software (SAS 9.3; SAS Institute Inc., Cary, NC, EUA, 2011). The following statistical model was applied for Experiment 1: Y_ijk_ = µ _+_ T_i +_ P(T_i_) _+_ b_1_ (w) + ε_ijk_.

Y_ijk_ = k_th_ observation of the i_th_ treatment (shade vs. no shade) of the j_th_ paddock.

µ = mean;

T_i_ = fixed effect of the treatment (shade vs. no shade);

P(T_i_) = random effect of the j_th_ paddock inside the i_th_ treatment;

b_1_ = linear regression coefficient for the initial weight;

ε_ijk_ = residual.

For Experiment 2, the statistical model was specified as follows: Y_ijkl_ = µ + T_i_ +P_j_ (T_i_ )+ G_k_ + TG_ik_ + b_1_ (w) + ε_ijkL_.

Y_ijkl_ = l_th_ observation of the i_th_ treatment (shade vs. no shade) of the j_th_ picket for the k_th_ breed.

µ = mean;

T_i_ = fixed effect of the treatment (shade vs. no shade);

P(T_i_) = random effect of the j_th_ picket inside the i_th_ treatment;

G_k_ = fixed effect of the breed;

b_1_ = linear regression coefficient for the initial weight;

ε_ijkl_ = residual.

## Results

### Meteorological variables

**Fig. 1:**
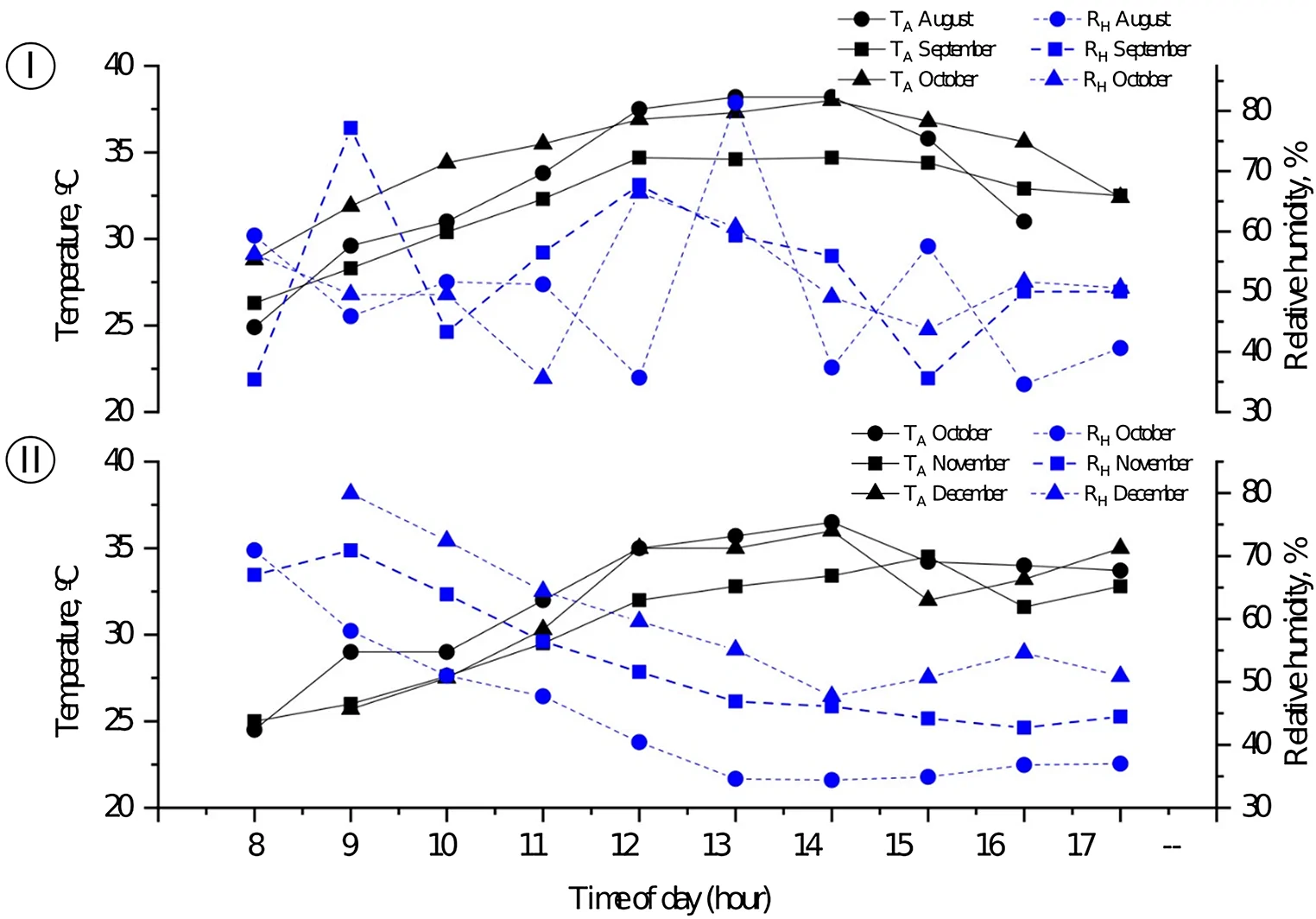
Meteorological variables measured in Experiments I and II throughout the day

Minimum, mean, and maximum values of the meteorological variables were recorded for both experiments (Table 3). Average air temperatures were 33.7 °C and 31.0 °C, while relative humidity averaged 48.9% and 51.5% in Experiments 1 and 2, respectively. In both experiments, the lowest temperatures were observed around 08:00, whereas the highest temperatures occurred around 14:00 (Fig. 1).

**Table 3:** Minimum, average, and maximum values of air temperature (TA), sun-exposed black-globe temperature (TG), and relative humidity (RH) recorded in Experiments 1 and 2

Variables	Minimum		Average		Maximum	
	Exp. I	Exp. II	Exp. I	Exp. II	Exp. I	Exp. II
TA (℃)	20.0	20.0	33.7	31.0	41.0	38.5
TG (℃)	22.0	27.0	43.4	44.1	57.0	56.0
RH (%)	23.7	22.0	48.9	51.5	91.7	100.0

### Water consumption

**Fig. 2:**
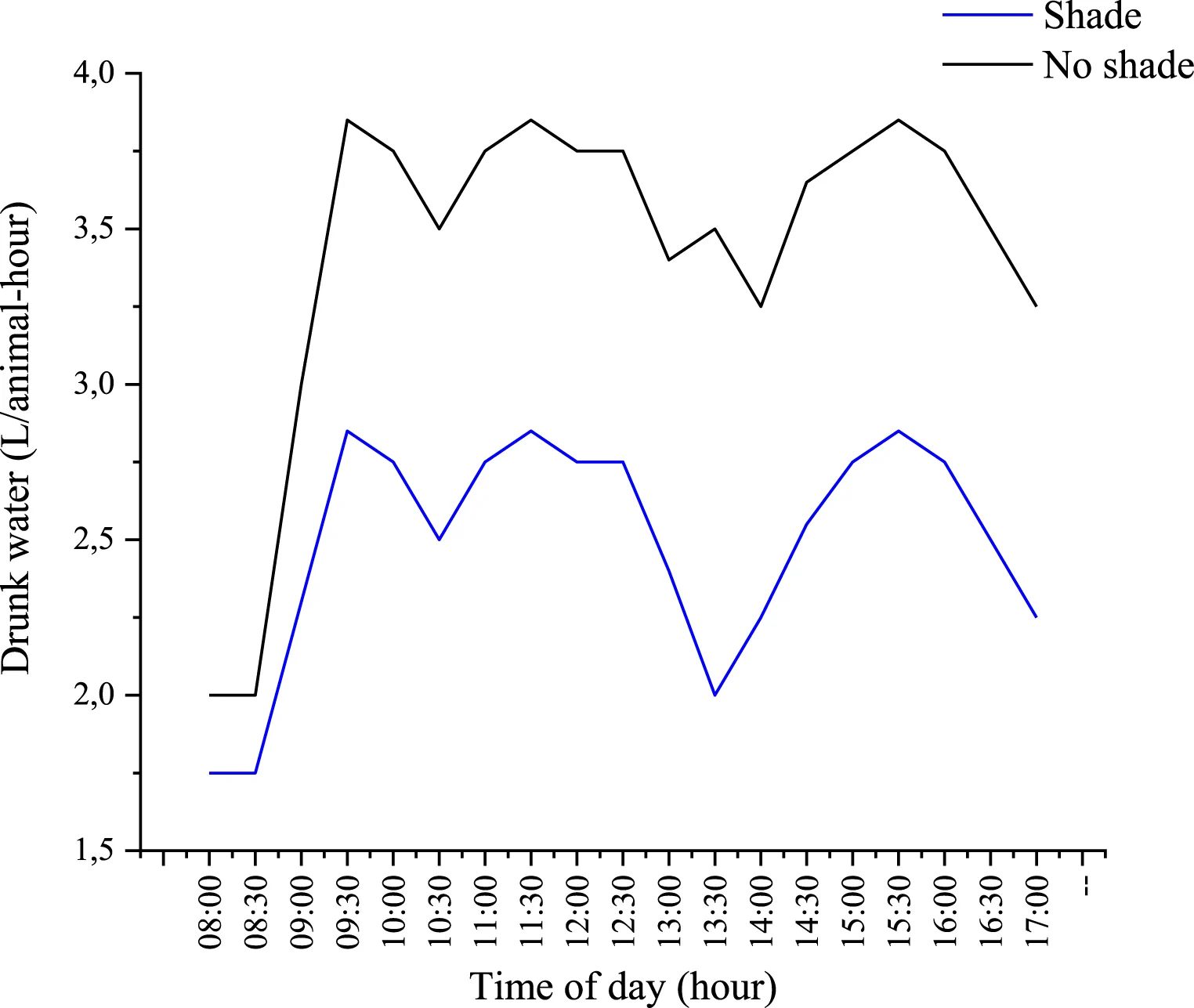
Hourly water intake of animals in Experiment II across the experimental period

During the experiments, water consumption was measured in animals with and without access to shade in Experiments 1 and 2. Animals provided with shade consumed less water than those without shade, suggesting lower thermal load (*P* < 0.05; 31.54 vs. 36.65 L/animal/day) (Fig. 2). Hourly and average daytime consumption (08:00–17:00) is presented for animals in both experiments, in paddocks with and without shade. Animals with access to shade consumed less than 3.0 L/animal/hour, whereas animals without shade consumed more than 3.5 L/animal/hour. Peak water intake occurred at similar times in both experiments and treatments, with the highest consumption observed at 09:30, 11:30, and 15:30.

### Performance

**Fig. 3:**
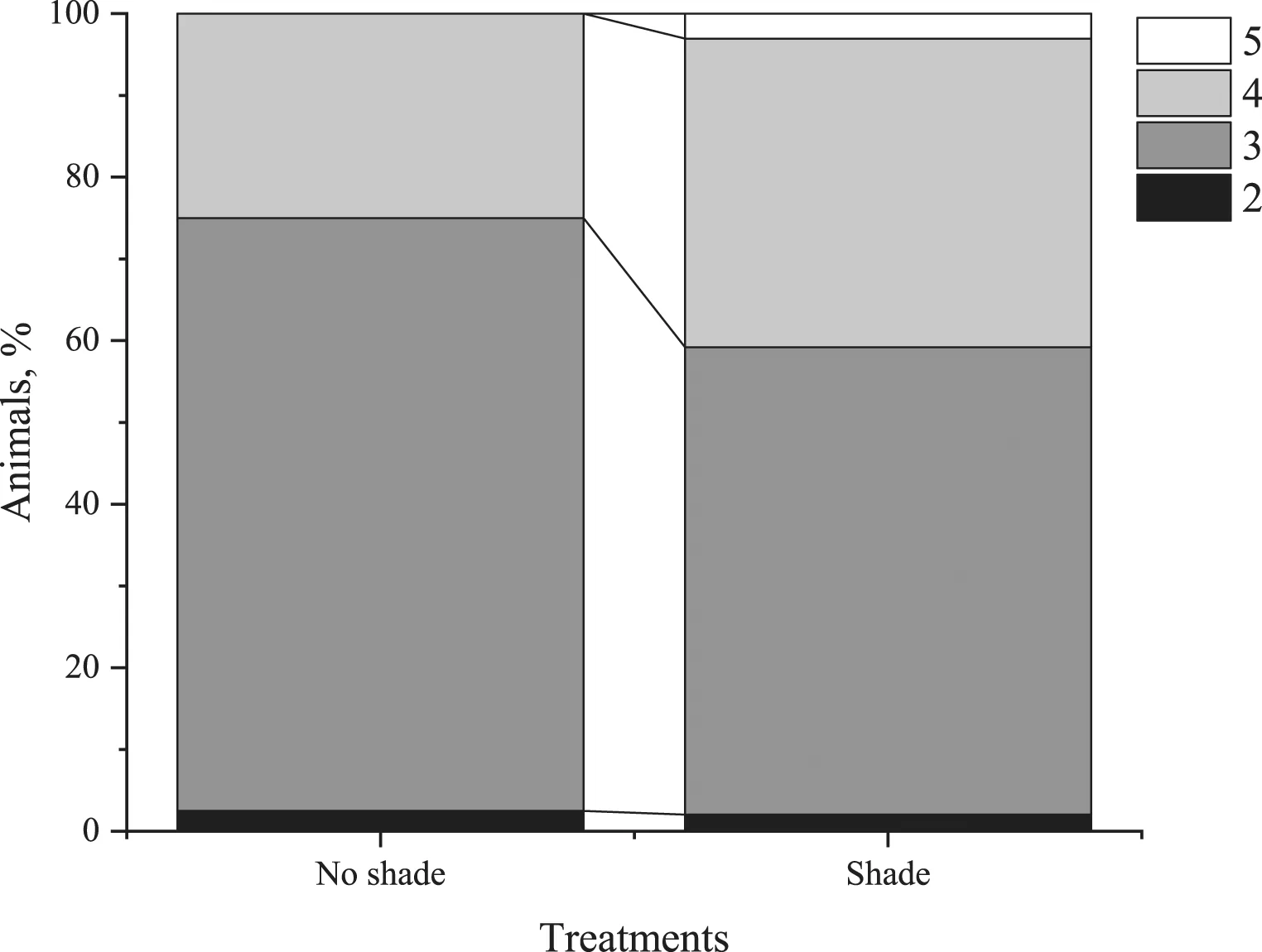
Distribution (%) of carcass finishing fat scores in animals with and without shade in Experiment I (2 = scarce, 3 = moderate, 4 = uniform, 5 = excessive)

Accumulated weight gain was 4.8% and 5.5% higher in animals with access to shade in Experiments II and I, respectively (*P* < 0.05). Carcass yield did not differ significantly between treatments. Finishing fat was greater in shaded animals (*P* < 0.05; 3.45 vs. 3.25; Table 4). Moreover, animals with access to shade exhibited greater uniformity in finishing fat scores (*P* < 0.05; Fig. 3), which is desirable from a commercial standpoint.

**Table 4:** Absolute mean values (± standard deviation) of performance variables—accumulated weight gain, carcass yield, and finishing fat—in Experiments I and II

Performance	Experiment	No Shade	Shade	*P*-value
Accumulated weight gain (kg)	I	85.99 ± 15.61	90.77 ± 19.40	0.03
	II	84.75 ± 17.62	88.85 ± 16.79	0.02
Carcass yield (%)	I	51.21 ± 2.51	51.57 ± 2.68	0.18
	II*	–	–	–
Fat in the carcass (index)	I	3.24 ± 0.49	3.45 ± 0.58	0.03
	II*	–	–	–

* carcass evaluations were not available from the commercial slaughter facility

## Discussion

Heat stress is a physiological condition induced by environmental factors (Navarini et al. 2009). The thermal comfort zone of beef cattle is reported to be up to 27 °C (Baêta and Souza 2010), while for Nellore cattle in shaded conditions it ranges from 24 to 35 °C (De Melo Costa et al. 2018). During the experiments, animals were exposed to environmental conditions characterized by high thermal load. Although physiological indicators of heat stress were not measured, the average THI values exceeded thresholds commonly associated with heat challenge in beef cattle. Brown-Brandl et al. (2017) suggested a THI (Temperature-Humidity Index) threshold of 74 as indicative of heat stress for beef cattle, whereas mean THI values of 83.13 and 80.06 were observed in Experiments I and II, respectively. Consistent with observations by Oliveira et al. (2019), under these environmental conditions, animals are more prone to seek shade to mitigate heat stress (Navarini et al. 2009). Providing shade can effectively reduce or eliminate heat stress, which is among the first measures recommended to protect animals from excessive heat load (Da Costa Ferro et al. 2016; Mishra 2021; Aranha, 2019).

Although the two experiments were conducted in different seasons, they were designed to evaluate the effects of artificial shade under two independent commercial feedlot systems operating according to their routine management schedules. While conducting both experiments during the same season would have facilitated direct comparisons, both experimental periods were characterized by high environmental thermal load, with mean THI values exceeding thresholds commonly associated with heat challenge in beef cattle. Therefore, despite the seasonal difference, the environmental conditions in both experiments were appropriate for evaluating the effects of shade under tropical production conditions.

Shade improves animal welfare by reducing radiant heat load on both the animal and the surrounding soil (Maia et al. 2023). Cooler soil temperatures facilitate heat dissipation from the animal and reduce heat gain via conduction and radiation, thereby alleviating heat stress (Kamal et al. 2015).

Higher water consumption in animals without shade is consistent with greater environmental thermal load and reduced thermal comfort. Although physiological evidence of heat stress was not collected, increased water intake has been reported as an adaptive response under hot environmental conditions. Increased water intake during high thermal load may result from plasma hyperosmolarity caused by evaporative heat loss through sweating and respiration, as well as increased urine output (McDowell and Weldy 1967). Central osmoreceptors detect hyperosmolarity and activate the hypothalamic thirst center in heat-stressed cattle (McDowell and Weldy 1967). By reducing water intake by approximately 5 L/animal/day, shading has the potential to save around 25 million liters of water daily, considering the scale of Brazilian beef production.

Artificial shading also improved productive performance, including higher accumulated weight gain and finishing fat, likely because shaded animals experienced a lower environmental thermal load, allowing a greater proportion of energy to be directed toward productive processes rather than thermoregulation. Similar results were reported by Aranha (2019), showing that shade increased weight gain and carcass yield in Nellore and crossbred animals. Gaughan et al. (2010) and Sullivan et al. (2011) also found that greater shade availability per animal improved carcass yield.

Several studies corroborate that providing shade during confinement enhances animal performance (Garcia Neto et al. 2016; Canozzi et al. 2022; Maia et al. 2023) and improves animal welfare, which is relevant both ethically and commercially in a market increasingly concerned with quality of life and sustainability (Moraes et al. 2020). Conversely, some studies reported no improvement in weight gain or carcass characteristics with shade (Dian et al. 2020; Ferreira et al. 2021). Novelli et al. (2022) found that while performance did not differ, shade reduced water requirements per unit of carcass produced, enhancing system efficiency.

In our study, average daily weight gain (≈ 950 g/day) was lower than in other studies (≈ 1,500 g/day), likely due to differences in feed type, animal genetics, and technological management. Shade quantity and quality also play a role; Maia et al. (2023) provided 3 m² of shade per animal with materials blocking solar radiation more effectively than 70% shade cloth.

Although dry matter intake was not measured in this study, increased environmental thermal load is known to reduce feed intake in cattle (Brown-Brandl et al. 2005; Beatty et al. 2006; Park et al. 2019) through the inhibition of hypothalamic nuclei involved in appetite regulation (Mishra 2021). Reduced feed intake decreases metabolic heat production but may also compromise weight gain. Nevertheless, the magnitude of this response depends on several factors, including breed thermotolerance, the intensity and duration of the thermal challenge, and environmental conditions during the night (Beatty et al. 2006; Pereira et al. 2008). Cooler nighttime temperatures allow cattle to dissipate the body heat accumulated during the day, providing a period of thermal recovery that can reduce the adverse effects of daytime heat load on feed intake and productive performance.

Bos indicus cattle are more efficient at heat dissipation, explaining the preference for zebu breeds in tropical Brazil, where they represent 42.6% of confined animals, with crosses accounting for an additional 34.1% (Scot Consultoria, 2021). Even with high thermotolerance, these animals respond positively to shade, emphasizing its importance.

Despite evidence of economic and environmental benefits (Novelli et al. 2022; Maia et al. 2023), the adoption of shade structures in commercial confinement remains limited, suggesting that this simple management practice is still underutilized.

## Conclusion

Providing artificial shade for confined beef cattle improved productive performance and reduced water consumption, which may indicate improved thermal comfort and contribute to better animal welfare under tropical feedlot conditions.

## Data Availability

The datasets generated and/or analysed during the current study are not publicly available because they contain information that could compromise farm privacy and animal management identities but are available from the corresponding author upon reasonable request.

## References

[CR35] Alvares CA, Stape JL, Sentelhas PC, de Moraes Gonçalves JL, Sparovek G (2013. Köppen’s climate classification map for Brazil. Meteorologische Zeitschrift 22(6):711–728. 10.1127/0941-2948/2013/0507

[CR1] Aranha H, Andrighetto C, Lupatini G, Bueno L, Trivelin G, Mateus G, Luz P, Santos J, Sekiya B, Vaz R (2019) Production and thermal comfort of Nellore beef cattle finished in integrated crop-livestock systems. Arquivo Brasileiro de Med Veterinária e Zootecnia 1686–1694. 10.1590/1678-4162-9913

[CR2] Baêta FC, Souza CF (2010) Ambiência em edificações rurais. Editora da UFV, Viçosa, p 246

[CR3] Beatty DT, Barnes A, Taylor E, Pethick D, McCarthy M, Maloney SK (2006) Physiological responses of Bos taurus and Bos indicus cattle to prolonged, continuous heat and humidity1. J Anim Sci 84:972–985. 10.2527/2006.844972x 16543576

[CR4] Brown-Brandl T, Eigenberg R, Hahn G, Nienaber J, Mader T, Spiers D, Parkhurst A (2005) Analyses of thermoregulatory responses of feeder cattle exposed to simulated heat waves. Int J Biometeorol 49:285–296. 10.1007/s00484-004-0250-2 15645291

[CR5] Brown-Brandl T, Chitko-McKown C, Eigenberg R, Mayer J, Welsh T, Davis J, Purswell J (2017) Physiological responses of feedlot heifers provided access to different levels of shade. Animal 11:1344–1353. 10.1017/S1751731116002664 28007043

[CR6] Canozzi MEA, Clariget J, Roig G, Pérez E, Aznárez V, Banchero G, La Manna A (2022) Shade effect on behaviour, physiology, performance, and carcass weight of heat-stressed feedlot steers in a humid subtropical area. Anim Prod Sci 62:1692–1705. 10.1071/AN22128

[CR7] Cooke RF et al (2020) Cattle adapted to tropical and subtropical environments: social, nutritional, and carcass quality considerations. J Anim Sci v 98:1–20. 10.1093/jas/skaa014 PMC7023624 31955200

[CR8] Cristhian Ferreira H, da Cunha Siqueira Carvalho C, Pinto Monção F, Júnior R, Mendes Ruas VR, da Costa JR, Antunes de Jesus MD, Magalhães Gonçalves M, Cristiane Mendes Rocha H, Ribas G (2021) Effect of shading strategies on intake, digestibility, respiratory rate, feeding behaviour, and performance of feedlot-finished Nellore bulls in the semi-arid region of Brazil. Italian Journal of Animal Science 20:1759–1769 10.1080/1828051X.2021.1912662

[CR9] Da Ferro C, Arnhold DA, Bueno E, Miyagi CP, Da Costa Ferro ES, dos Santos RA, dos Santos APP, K.J.G., and, da Silva BPA (2016) Physiological and behavioral responses of Nellore steers to artificial shading in an intensive production system. Semina: Ciências Agrárias 37:2785–2792. 10.5433/1679-0359.2016v37n4Supl1p2785

[CR10] De Felicio PE (1981) Carcass composition and quality traits of zebu steers slaughtered in the state of São Paulo, Brazil. Kansas State University

[CR11] De Melo Costa CC, Maia ASC, Nascimento ST, Nascimento CCN, Neto MC, de França Carvalho Fonsêca V (2018) Thermal balance of Nellore cattle. International Journal of Biometeorology 62:723–731 10.1007/s00484-017-1349-6 28421269

[CR12] Dian PHM, dos Santos MCB, de Andrade Belo MA, Brennecke K, de Melo GMP, de Melo WJ (2020) Desempenho zootécnico e financeiro de bovinos confinados com acesso a diferentes áreas de sombreamento e a pleno sol. Brazilian J Dev 6:101646–101664. 10.34117/bjdv6n12-599

[CR13] Garcia Neto S, Nascimento J, Matos Junior J, Silva L, Meira A (2016) Desempenho de bovinos mestiços criados em confinamento com disponibilidade de sombreamento natural e artificial. In: Congresso Técnico Científico da Engenharia e da Agronomia, Foz do Iguaçu, PR, p 5

[CR14] Gaughan J, Bonner S, Loxton I, Mader T, Lisle A, Lawrence R (2010) Effect of shade on body temperature and performance of feedlot steers. J Anim Sci 88:4056–4067. 10.2527/jas.2010-2987 20709874

[CR15] Gaughan jb, Lees am, Lees JC (2021) Adaptation of Beef Cattle to Heat Stress Challenges. In: SEJIAN V et al (eds) Climate Change and Livestock Production: Recent Advances and Future Perspectives. Springer, Singapore, pp 69–87. 10.1007/978-981-16-9836-1_3

[CR16] Kamal R, Dutt T, Patel M, Dey A, Chandran PC, Bharti PK, Barari SK (2015) Behavioural, biochemical and hormonal responses of heat-stressed crossbred calves to different shade materials. J Appl Anim Res 44:347–354. 10.1080/09712119.2015.1074076

[CR17] Lotti T, Ferrarezi JE (2023) Bem-estar animal na produção do gado de corte: uma revisão bibliográfica. Revista Interface Tecnológica 20(2):690–699. 10.31510/infa.v20i2.1708

[CR18] Maia ASC, Culhari EdA, Fonsêca VdFC, Milan HFM, Gebremedhin KG (2020) Photovoltaic panels as shading resources for livestock. J Clean Prod 258:120551. 10.1016/j.jclepro.2020.120551

[CR19] Maia ASC, Moura GAB, Fonsêca VFC, Gebremedhin KG, Milan HM, Chiquitelli Neto M, Simão BR, Campanelli VPC, Pacheco RDL (2023) Economically sustainable shade design for feedlot cattle. Front Veterinary Sci 10. 10.3389/fvets.2023.1110671 PMC9905632 36761885

[CR20] McDowell RE, Weldy JR (1967) Water Exchange of cattle under heat stress, In: Proceedings of the 3rd International Biometeorological Congress, London, UK, 414–424

[CR21] Mishra S (2021) Behavioural, physiological, neuro-endocrine and molecular responses of cattle against heat stress: an updated review. Trop Anim Health Prod 53:400. 10.1007/s11250-021-02790-4 34255188

[CR22] Moraes ER, Ishihara JH, Sales DE (2020) Efeito do bem-estar e conforto térmico na produção pecuária: uma revisão bibliográfica. Res Soc Dev 9. 10.33448/rsd-v9i9.7913

[CR23] Navarini FC, Klosowski ES, Campos AT, Teixeira RdA, Almeida CP (2009) Conforto térmico de bovinos da raça nelore a pasto sob diferentes condições de sombreamento e a pleno sol. Engenharia Agrícola 29:508–517. 10.1590/S0100-69162009000400001

[CR24] Novelli TI, Bium BF, Biffi CHC, Picharillo ME, de Souza NS, de Medeiros SR, Palhares JCP, Martello LS (2022) Consumption, productivity and cost: Three dimensions of water and their relationship with the supply of artificial shading for beef cattle in feedlots. J Clean Prod 376:134088. 10.1016/j.jclepro.2022.134088

[CR25] Oliveira SEO, de Melo Costa CC, Chiquitelli Neto M, Dalla Costa FA, Maia ASC (2019) Effects of shade location and protection from direct solar radiation on the behavior of Holstein cows. Int J Biometeorol 63:1465–1474. 10.1007/s00484-019-01747-5 31254070

[CR26] Palhares JCP et al (2023) Economically sustainable shade design for feedlot cattle. Frontiers in Veterinary Science 9 Disponível em: 10.3389/fvets.2023.1110671. Acesso em: 05 out. 2025 PMC9905632 36761885

[CR27] Park JH, Choi HC, Lee HJ, Kim ET, Son JK, Kim DH (2019) A Study on the Effect of Temperature-Humidity Index on the Respiration Rate, Rectal Temperature and Rumination Time of Lactating Holstein Cow in Summer Season. J Korea Academia-Industrial Cooperation Soc 20:136–143. 10.5762/KAIS.2019.20.11.136

[CR28] Park RM, Foster M, Daigle CL (2020) A scoping review: the impact of housing systems and environmental features on beef cattle welfare. Animals 10:565. 10.3390/ani1004056532230891 PMC7222360

[CR29] Pereira AM, Baccari F, Titto EA, Almeida JA (2008) Effect of thermal stress on physiological parameters, feed intake and plasma thyroid hormones concentration in Alentejana, Mertolenga, Frisian and Limousine cattle breeds. Int J Biometeorol 52:199–208. 10.1007/s00484-007-0111-x 17578605

[CR30] SAS Institute Inc (2011) SAS 9.3, SAS 9.3 Documentation by Title - SAS Support. SAS Institute Inc, Cary, NC

[CR31] Scot Consultoria. In: Notícias. Benchmarking Confina Brasil (2021) https://www.scotconsultoria.com.br/noticias/todas-noticias/54872/. Accessed 29 May 2023

[CR32] Silveira GM, Leme DP, Romero RB (2020) Cattle adapted to tropical and subtropical environments: social, nutritional, and carcass quality considerations. Tropical Animal Health and Production, v. 52, n. 1, pp. 13–18. 10.1007/s11250-019-02052-7 PMC7023624 31955200

[CR33] Sullivan M, Cawdell-Smith A, Mader T, Gaughan J (2011) Effect of shade area on performance and welfare of short-fed feedlot cattle. J Anim Sci 89:2911–2925. 10.2527/jas.2010-3152 21478450

[CR34] Wankar AK et al (2024) Heat stress in beef cattle: climate change and the global scenario–a review. Annals Anim Sci v 24(4):1093–1105. 10.2478/aoas-2024-0010

